# Cerebral Oxygenation Is Highly Sensitive to Blood Pressure Variability in Sick Preterm Infants

**DOI:** 10.1371/journal.pone.0043165

**Published:** 2012-08-14

**Authors:** Flora Y. Wong, Reshma Silas, Simon Hew, Thilini Samarasinghe, Adrian M. Walker

**Affiliations:** 1 The Ritchie Centre, Monash University, Melbourne, Victoria, Australia; 2 Monash Newborn, Monash Medical Centre, Melbourne, Victoria, Australia; 3 Department of Paediatrics, Monash University, Melbourne, Victoria, Australia; University of Giessen Lung Center, Germany

## Abstract

**Objectives:**

The significance of blood pressure variability (BPV) for cerebral oxygenation in extremely preterm infants has not been explored, though BPV may well be associated with end organ injury. We hypothesized that increased BPV in sick preterm infants, by exceeding the cerebral autoregulatory capacity, is associated with cerebral oxygenation changes which closely follow the blood pressure fluctuations. We assessed the autoregulatory capacity in the early postnatal period, by determining the correlation between BPV (mmHg^2^) and coherence of mean arterial blood pressure (MABP mmHg) and cerebral oxygenation (tissue oxygenation index, TOI %).

**Study Design:**

Thirty-two preterm infants of mean gestational age of 26.3 (±1.5) weeks were studied on the first 3 postnatal days. Spectral analysis (Coherence and transfer-function gain analysis) was used to calculate coherence of MABP and TOI; BPV was quantified using power spectral density of MABP.

**Results:**

Overall, maximum Coherence showed a trend for positive correlation with BPV (n = 32, p = 0.06). Infants identified as clinically unstable with documented brain injury (n = 7) had high Coherence values at low BPV. Separate analysis of stable infants (excluding the 7 critically ill infants) revealed a significant association between maximum Coherence and BPV (n = 25, p = 0.006).

**Conclusions:**

Fluctuation in cerebral oxygenation is closely associated with increased BPV in preterm infants undergoing intensive care. Moreover, in the critically sick preterm infant, blood pressure-dependent variations in cerebral oxygenation occur even with relatively lower BPV, suggesting they have severely impaired autoregulation, and placing them at greater vulnerability to cerebral injury arising from blood pressure fluctuations.

## Introduction

Cerebral periventricular haemorrhage and white matter injury are most likely responsible for the majority of neurological deficits in survivors of preterm birth [1]. The cerebral white matter is at increased risk of injury due to intrinsic vascular and physiological factors, with arterial end zones sensitive to falls in cerebral perfusion [2], and also relatively low regional cerebral blood flow (CBF) [3], [4]. In the healthy mature brain, cerebral autoregulation limits CBF variation over a range of cerebral perfusion pressures, so ensuring adequate perfusion and oxygenation of the brain. However, in the preterm animal, the resting blood pressure is close to the lower limits of autoregulatory capacity [5], and several reports in sick preterm infants have also demonstrated limited or impaired autoregulation [6], [7], so that CBF fluctuates with changes in arterial blood pressure. Under such circumstances, the degree of blood pressure variability (BPV) may be clinically important, should large fluctuations in blood pressure exceed autoregulatory capacity and result in hyperemia and/or ischemia in the vulnerable arterial end zones of the germinal matrix and cerebral white matter**.** In support of the potential adverse effect of high BPV on preterm brain injury, high BPV in hypertensive adults has been reported to be an important determinant of end organ injury [8].

Cerebral oxygenation and autoregulation in sick preterm infants have been assessed using Spatially resolved spectroscopy (SRS) [9], which measures a tissue oxygenation index (TOI, %). Cerebral TOI reflects a mixed oxygen saturation but is most influenced by the venous compartment, which represents ∼75% of total cerebral blood volume. Assuming constant cerebral metabolic rate for oxygen within a relatively short period of time, changes in cerebral venous oxygen saturation and, therefore, changes in TOI will parallel variations of CBF according to the Fick principle. If autoregulation fails, TOI, as a surrogate for CBF, should follow fluctuations in mean arterial blood pressure (MABP) [9], [10]. The degree of correlation of TOI and MABP can therefore be used to quantify cerebral autoregulation [9].

In this study, we aimed to determine the relationship between cerebral autoregulatory capacity and BPV in extremely preterm infants. To assess the implications for injury we focused on the first 3 postnatal days when most intraventricular haemorrhages (IVH) occur in preterm infants [11]. We applied cross-spectral analyses (coherence and transfer function) to quantify the frequency-dependent covariation of TOI and MABP as a means of assessing autoregulation. Blood pressure variability was quantified as the power spectral density of MABP. We hypothesized that in sick preterm infants an increased BPV would exceed the cerebral autoregulatory capacity, leading to high coherence between TOI and MABP.

## Methods

### Ethics

This project had approval from Monash Medical Centre Human Research Ethics Committee and written parental informed consent was obtained prior to each study.

### Participants

Preterm infants were eligible if they were born at ≤29 weeks gestational age, with a birth weight ≤1000 gm, and an intra-arterial catheter. Infants with major congenital abnormalities were excluded.

Thirty-two extremely low birthweight infants (Table 1) were studied for 4–6 hours a day for the first 3 postnatal days. Twenty-three of the 32 infants were successfully studied on all 3 days. Incomplete studies were largely due to mechanical factors such as the loss of arterial lines. The mean (±SD) postnatal age at the time of study on days 1–3 was 12(±5.8) hr, 35(±6.1) hr and 57(±5.9) hr respectively. Clinical management of the infants was at the sole discretion of the attending neonatologist. During the study period, ten infants were given inotropic medications (dobutamine, n = 2; dopamine, n = 7; dobutamine and dopamine, n = 1; dobutamine and dopamine and adrenaline, n = 1) for hypotension, defined and treated to maintain the MABP in mmHg at more than or equal to the gestational age in weeks [12]. None of the infants was considered to be hypotensive during the recordings. Cranial ultrasounds to identify and categorize IVH [2] were performed daily on the 3 days of study.

**Table 1:** Characteristics of the infants (n = 32).

Infant Characteristic	Values
Gestational age (weeks, mean±SD)	26.3±1.5
Birth weight (g, mean±SD)	837±170
Male (n)	17
Female (n)	15
Mechanical ventilation (n)	32
Inotrope medication (n)	10
Significant findings on cerebral imaging*	5
Clinical Risk Index for Baby Score (mean±SD)	5.1±3.3
Death (n)	1

* Grade 3–4 intraventricular haemorrhage on ultrasound or MRI evidence of severe hypoxic-ischaemic brain injury.

Seven infants were clinically extremely unwell during the study, and subsequently had documented brain injury or died. These infants included one who died on day 3 due to systemic embolism secondary to pulmonary interstitial emphysema, and five who sustained severe cerebral injury (One infant with bilateral grade III IVH, 1 infant with bilateral grade III and progressed to hydrocephalus requiring VP shunt with trigonal white matter loss on MRI at term, 2 infants with grade IV IVH, and 1 infant with perinatal hypoxic ischemic encephalopathy and severe brain injury shown on MRI). One additional infant had severe pulmonary hemorrhage with resultant profound hypoxemia and severe metabolic acidosis during the study; the MRI at term-corrected age showed loss of white matter in the periventricular region. The cranial ultrasound findings in the other 25 infants ranged from normal (n = 16), to grade I (n = 7) or grade II (n = 2) IVH.

### Measurements

#### Tissue oxygenation index

Spatially resolved spectroscopy (NIRO 200; Hamamatsu Photonics K.K., Hamamatsu City, Japan) was employed for continuous TOI recording. Two aligned photodetectors are housed inside the detection probe, fixed at 4 cm from the emission probe. The probes were placed over the temporo-parietal region of the infants. The SRS algorithm [13] measures TOI which was logged at 6 Hz.

#### Physiological measurements

Continuous measurements of MABP were obtained from an indwelling intra-arterial catheter connected to a transducer monitoring system (Transpac IV, Abott Australasia, Kurnell, NSW, Australia). The blood pressure waveforms and values, along with the continuously monitored transcutaneous partial pressure of carbon dioxide (PaCO_2_) were displayed on a cotside monitor. Arterial oxygen saturation (SaO_2_) was obtained via an oximeter (Nellcor N200, Nellcor Inc, Hayward, California, USA) from an oximetry probe secured to the right upper limb.

#### Data acquisition and collection

Analogue signals of the cotside physiologic data were collected and digitised at 400 Hz (PowerLab 16SP, ADInstruments, NSW, Australia), and were then down- sampled to 6 Hz to allow for offline spectral analysis with TOI which was collected simultaneously. Uninterrupted simultaneous recordings of TOI and MABP, with stable PaCO_2_ (less than 8 mmHg fluctuation) and SaO_2_ (less than 5% fluctuation), were divided into 20-minute segments for subsequent analysis. TOI recordings were excluded from analysis if movement artifacts caused a baseline shift of ≥10%. In practice this excluded recordings during spontaneous head movements, physical examinations, radiographs, clinical procedures, and other events requiring handling of the infant. Segments were also excluded if arterial pressure recordings were interrupted by blood gas sampling or limb movements when the intra-arterial line was in a peripheral limb site/placement.

#### Data analyses

Correlation between continuous measurements of MABP and TOI were quantified in a frequency-specific manner by established spectral analysis techniques [14]. Computations were performed (MatLab v7.11; MathWorks, Inc, Natick, MA) on 20-minute epochs as previously described [9]. Power spectral densities (PSDs, Welch’s method, MatLab) of TOI and MABP [P_TOI_(f) and P_MABP_(f)], and their cross PSD [P_TOI,MABP_(f)] were used to generate the coherence function for the two signals |P_TOI,MABP_(f)|^2^/[|P_TOI_(f)| |P_MABP_(f)|], and their transfer function, P_TOI,MABP_(f)/P_MABP_(f).

The coherence function characterizes the frequency dependent correlation of MABP with TOI. The numeric value of the coherence function (Coh) at a given frequency ranges between zero and 1.0; Coh values of ≥0.5 are considered to represent significant frequency-specific concordance between the 2 waveforms [14]. High concordance between TOI and MABP and, therefore, high Coh values, can be anticipated when autoregulation is impaired and changes in TOI parallel those of MABP. BPV was obtained from the PSD [P_MABP_(f)] of MABP.

The transfer function gain [(G), % per mmHg] represents the magnitude of change in the output (TOI) arising from a unity change in the input (MABP) at a given frequency. Impaired autoregulation, signified by a large magnitude change of TOI for a nominal change of MABP, gives rise to a high magnitude for G. G values were used for analysis only if the corresponding Coh of the 20-minute segment was ≥0.5, since at Coh <0.5, the fundamental assumption for linear transfer function gain may be violated [15].

Coh, BPV and G values were averaged for frequencies between 0.003 and 0.02 Hz. The upper limit was chosen to be 0.02 Hz as autonomic vascular control of cerebral autoregulatory processes occur at frequencies below this level [16]. The lower limit of 0.003 Hz was chosen to be above the lower frequency limit which was determined by the data segment length of 20 minutes.

The correlation between Coh and BPV was analysed by selecting the highest Coh value on each of the 3 postnatal days, and also the overall maximum Coh value over the entire 3 postnatal days. We selected these peak values of Coh as representative of periods of greatest autoregulatory impairment which may place the infant at greatest clinical risk of cerebral injury [17].

For infants with maximum Coh ≥0.5, the corresponding G values were compared between infants of different degree of clinical sickness.

### Statistical Methods

Sequential data analysis was performed (Sigma Stat v3.5, SPSS Inc, Chicago, USA) to evaluate the relationship between Coh and BPV. Firstly, Analysis of Covariance (ANCOVA) [18] was used to quantify the correlation between Coh and BPV within infants with multiple 20-minute segments. Secondly, linear regression and Pearson’s Correlation Coefficient were used to examine the relationship between the highest Coh for each infant on each postnatal day and the corresponding BPV, and thirdly, between the maximum Coh for each infant on any of the 3 postnatal days with the corresponding BPV. Student’s *t* test was used to determine the difference between BPV for infants receiving inotropic support and those who were not, and also to determine the difference in TOI values between the 7 critically sick infants and the rest of the infants. The Mann-Whitney Rank Sum test was used to determine the difference in the median values of G between the 7 critically sick infants and the rest of the infants, as the data did not show a normal distribution. One Way Repeated Measures ANOVA was used to determine change in MABP and TOI over the 3 days. A P-value of less than 0.05 was considered statistically significant.

## Results

### Blood Pressure Variability

**Figure 1:**
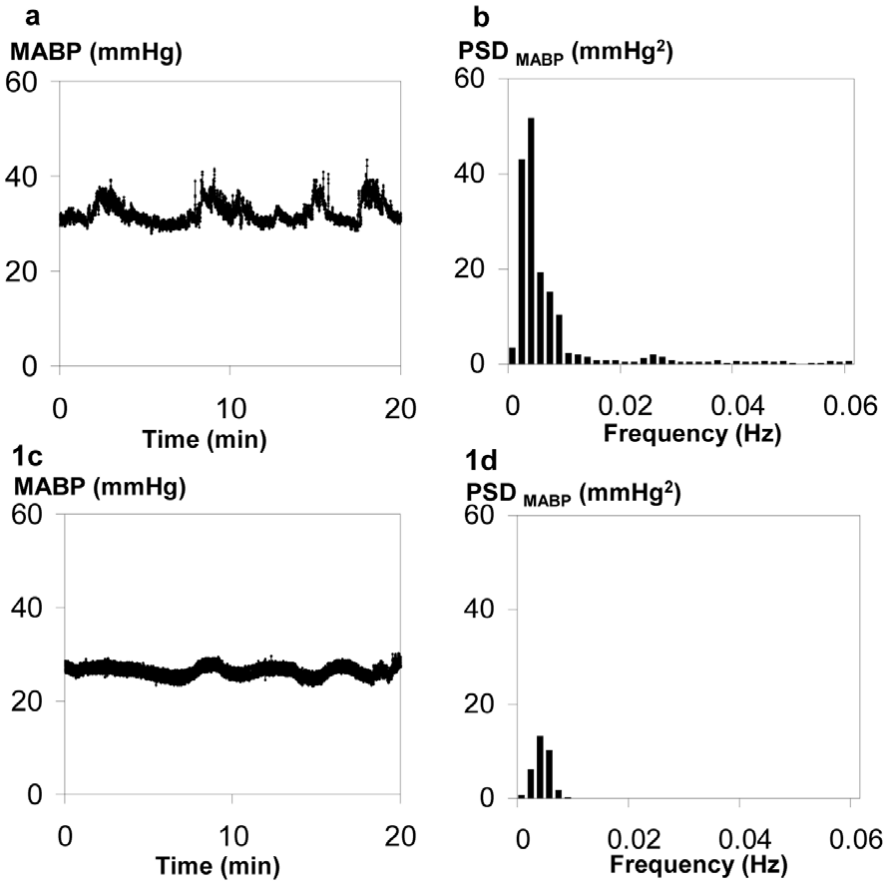
MABP waveform and its Power Spectral density (PSD). (a) Highly fluctuant MABP waveform over 20 minutes; (b) Power Spectral density (PSD) of MABP from (a) with averaged PSD of 9.6 mmHg^2^ in the frequency range of 0.003–0.02 Hz, signifying high BPV; (c) Minimally fluctuant MABP waveform; (d) PSD of MABP from (c) averaged between 0.003–0.02 Hz is 2.3 mmHg^2^, indicating low BPV.

Figure 1 illustrates the concordance of MABP variation in time and frequency domains. Large MABP fluctuations clearly evident in the time domain (Figure 1a), are also expressed as high MABP variability in the frequency domain, as shown in the power spectrum (Figure 1b). In contrast, a MABP waveform with small fluctuations in the time domain shows correspondingly low power (Figure 1c and d).

### Coherence

**Figure 2:**
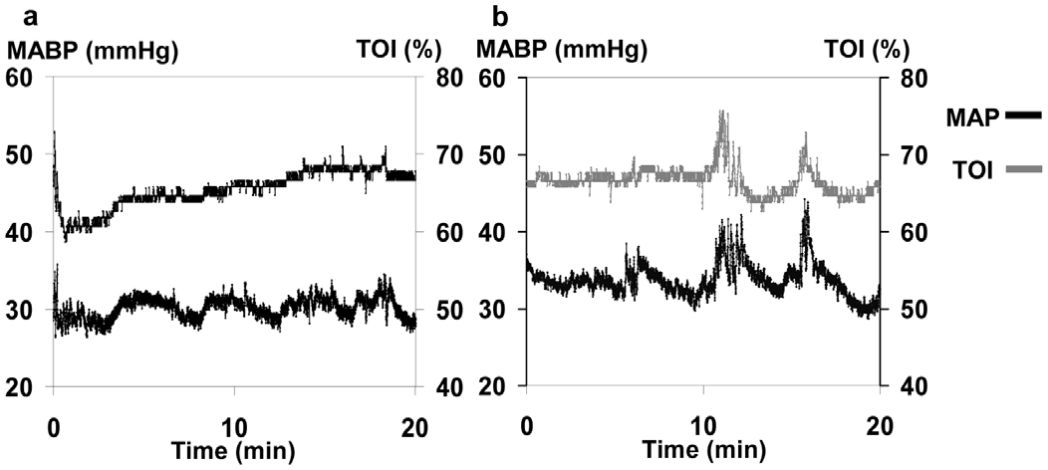
Coh between MABP and TOI. (a) Low Coh between MABP and TOI. Despite fluctuations in MABP, TOI remains relatively unchanged, indicating intact autoregulation. The Coh value in the 0.003–0.02 Hz frequency range is 0.40. (b) High Coh between MABP and TOI. Note the concordant fluctuations in MABP and TOI waveforms suggesting impaired autoregulation; Coh is 0.66.

Figure 2 illustrates two 20-minute segments of MABP and TOI recordings. Figure 2a depicts changes in MABP which are not accompanied by TOI changes; cross spectral analysis showed a low Coh of 0.40. In contrast, in Figure 2b changes in MABP are accompanied by concordant changes in TOI, with a significant Coh of 0.66.

### Coherence and Blood Pressure Variability

The mean (± SD) numbers of 20-minute segments free of movement artefacts per infant used for analysis, were 5(±1.6), 5.1(±1.6) and 5(±1.8) on postnatal days 1–3 respectively. The averaged MABP and TOI for the infants on postnatal days 1–3 are shown in Table 2, with a significant increase in MABP on D3 compared to D1, while the change in TOI did not reach significance. On the three postnatal days, TOI values for 7 sick infants were 62.1±6.3%, 64.2±9.3% and 62.4±6.2% respectively, lower than the averaged TOI values for the rest of the infants (63.8±5.8%, 65.0±6.4% and 67.5±4.4% respectively); the difference reached significance on D3 (P = 0.03). The TOI and PCO_2_ values averaged for each postnatal day in all infants showed a positive correlation (R^2^ = 0.052, P = 0.04), while the TOI and SaO_2_ values averaged for each postnatal day showed a trend for positive correlation (R^2^ = 0.025, P = 0.08).

**Table 2:** Correlation within infants (ANCOVA) between Coherence (Coh, MABP with TOI) and Blood pressure variability (BPV) for postnatal days 1, 2 and 3.

Postnatal days	Number of infants (N)	MABP (mmHg, mean±SD)	TOI (%, mean±SD)	ANCOVA between Coh and BPV R^2^ p-value
D1	29	32.4 (±4.3)	63.4 (±5.8)	0.12 0.03*
D2	26	33.4 (±4.1)	64.8 (±7.0)	0.01 0.28
D3	23	35.1 (±5.3)#	66.3 (±5.2)	0.19 0.01*

* p<0.05.

# p<0.05 compared to D1.

**Figure 3:**
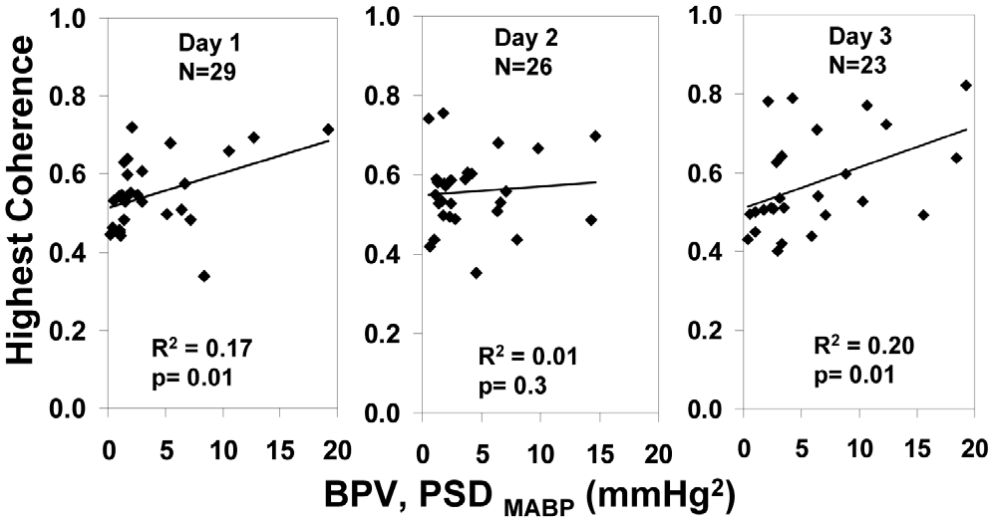
Correlation between highest daily Coh (MABP with TOI) and BPV for each postnatal Day 1–3. Note relationship is significant on postnatal Days 1 and 3.

The correlation between Coh and BPV within infants (ANCOVA, Table 2) was significant on postnatal days 1 and 3. The highest Coh, indicating the highest degree of TOI-MABP covariation, obtained for each infant on each postnatal day is plotted against the corresponding BPV (Figure 3); a significant correlation was evident on postnatal days 1 and 3, consistent with the correlations from ANCOVA (Table 2).

**Figure 4:**
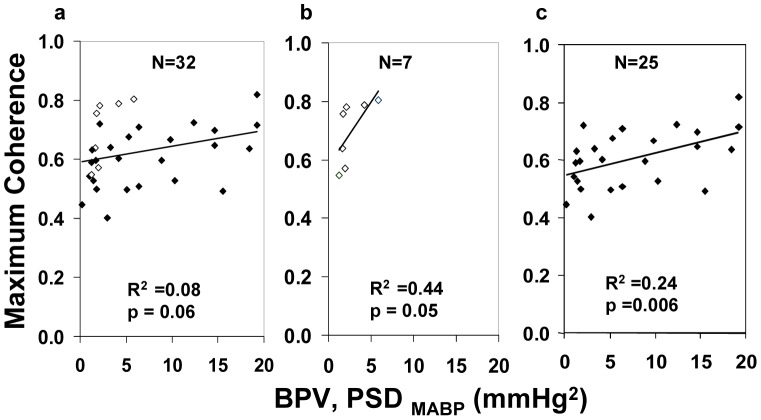
Correlation between the Maximum Coh (MABP with TOI) and BPV (a) Trend for positive correlation for all infants: ◊ 7 sickest infants; ♦ stable infants. (b) ◊ sickest infants show high Maximum Coh with relatively low BPV. (c) ♦ stable infants with sickest infants excluded exhibit a significant positive relationship.

The single maximum Coh of each infant over the entire 3 days showed a trend towards positive association with the corresponding BPV (R^2^ = 0.08, P = 0.06, Figure 4a). Seven critically unwell infants, who had significant cerebral injury or died, all exhibited a high Coh at relatively low BPV, with positive association between Coh and BPV (Figure 4b). On exclusion of these 7 infants from the analysis, a statistically significant association was revealed for the remaining infants (R^2^ = 0.24, P = 0.006, Figure 4c). MABP associated with maximum Coh of the 7 critically unwell infants was not different from that of the remainder (34.5 (±4.2) and 34.1(±5.2) mmHg respectively). In the 4 infants who developed Grade 3–4 IVH, the maximum Coh values over the entire 3 days all occurred on the day immediately before the IVH developed.

Median (IQR) G associated with maximum Coh was higher in 7 critically unwell infants compared with the rest of the infants in the study [0.59 (0.48–1.0) vs. 0.39 (0.31–0.61) %/mmHg, (P = 0.03)]. BPV for 20-minute segments associated with ongoing inotropic support was not significantly different from those without support, with values of 3.31(±4.17) and 3.97(±4.49) mmHg^2^ (P = 0.14) respectively. Of the 10 infants who received inotropic support, 2 developed Grade III-IV IVH; in these infants there was no evident relationship between their level of hypotension, the institution of inotropic support and the development of moderate to severe IVH.

## Discussion

To our knowledge this is the first study in extremely preterm infants to employ spectral analysis to examine the relationship between fluctuations in cerebral oxygenation and BPV that takes account of the specific clinical status of the infants. Overall, our results revealed a significant positive association between the degree of coherence of cerebral TOI with MABP (Coh), and the extent of blood pressure variability (BPV) on the first and third postnatal days. In clinically stable infants, a significant correlation between the maximum Coh and BPV emerged after exclusion of those infants with cerebral injury or who were clinically very unwell, suggesting that the form of relationship between Coh and BPV may be altered in infants who are critically ill with low TOI values. As increased Coh indicates cerebral autoregulatory impairment [9], our finding implies that in the clinically stable infants, a pressure-passive cerebral circulation may occur with high BPV which exceeds autoregulatory capacity. By contrast, in critically unwell infants, high coherence signifying severely impaired autoregulation and pressure passive perfusion may occur even with relatively low blood pressure fluctuations. Notably, in the infants who developed Grade III/IV IVH, maximum Coh values all occurred on the day immediately before the IVH developed, suggesting a role of impaired autoregulation on the development of the IVH.

Though overall physiological relationships were evident between TOI, SaO_2_ and PaCO_2_, the Coh values and the relationships between Coh and BPV do not reflect these associations, as all coherence analyses for MABP and TOI were conducted within infants using data segments with stable PaCO_2_ and SaO_2._ Technically, increased Coh could be evident at higher BPV due to the improved ‘signal-to-noise’ ratio at higher BPV [19]. However, the high Coh seen even at low BPV in the critically unwell infants suggests the finding has a biological basis rather than a methodological one. Lending further support to the presence of severe autoregulatory impairment in critically unwell infants, G values are high, signifying high magnitude TOI fluctuations for a given MABP change.

Limited or impaired cerebral autoregulation in sick preterm infants has been shown in previous studies [6], [7], though individual studies have suggested different MABP values as the lower limit of the autoregulatory plateau below which CBF decreases with further reduction in MABP [20], [21]. The upper limit of the autoregulatory curve for preterm infants, however, has not been determined. Possibly, the “plateau” of the autoregulatory curve may have a small positive slope and the range and position of the thresholds may vary between infants [21]. Furthermore, there may also be periodic variation in autoregulatory capacity within individual preterm infants [22]. Such variations in autoregulatory capacity between and within infants may have contributed to our findings of varying associations between Coh and BPV in Days 1–3, specifically the apparent absence of correlation on Day 2. Importantly our results (Figure 4) reveal that clinical conditions of infants may affect the autoregulatory capacity, with critically unwell infants showing poor autoregulation with relatively low BPV. Consistent with our results, previous reports also showed that clinical conditions may influence the autoregulatory capacity in sick newborns [7], [23]. Interestingly, the median value of G of 0.59%/mmHg in the critically sick infants translates to ∼0.3% change in TOI per % change in MABP. As the figure is below the 1% change in TOI per % change in MABP which would represent a completely pressure-passive cerebral circulation, our results may indicate some residual autoregulation even in the sickest infants.

**Figure 5:**
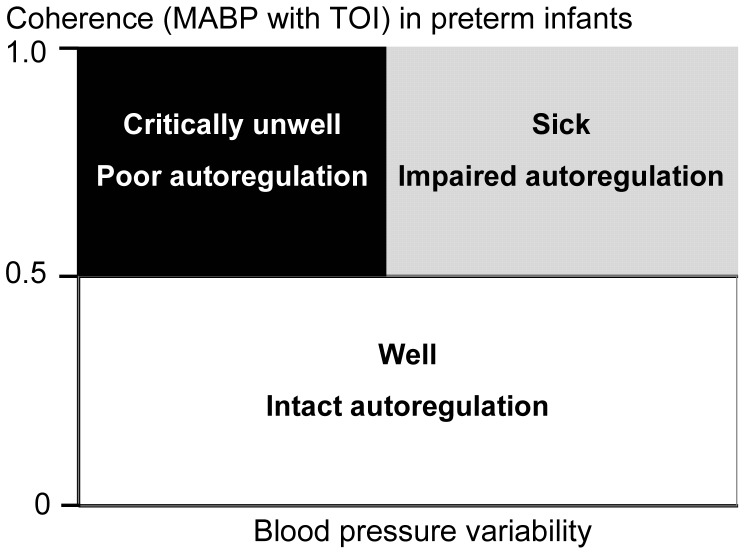
Schematic model of cerebral autoregulation versus clinical condition of preterm infants. Well infants with intact autoregulation have Coh<0.5 over a wide range of BPV. Sicker infants with impaired autoregulation have Coh>0.5 when BPV is high and exceeds autoregulatory capacity. Sickest infants with absent autoregulation have Coh>0.5 even with relatively low BPV.

The association between Coh and BPV in relation to the clinical stability of the very preterm infant is summarized schematically in Figure 5. We propose that the well preterm infant autoregulates cerebral perfusion over a wide range of BPV, and has a low Coh that remains <0.5 despite high BPV. Similarly, an unwell preterm infant is capable of maintaining cerebral autoregulation and exhibits a low Coh provided BPV remained relatively low; however, larger BPV would exceed such an infant’s autoregulatory capacity, causing fluctuation of cerebral oxygenation with changes in blood pressure, resulting in high Coh. Finally, the critically unwell infant autoregulates poorly and exhibits high Coh even at low BPV.

The factors underlying different degrees of BPV in preterm infants have not been studied. Normal BPV arises from the interaction between external stimuli and the regulatory mechanisms involved with behavioural variations, respiration and cardiovascular control. High frequency oscillations in BP are related to the effect of respiration on cardiac output and also by sympathetic control of vasomotor tone [24]. However the mechanisms responsible for the very low frequency oscillations in BP which are important in our study are largely unknown. It is possible that they may be related to clinical environmental factors, for example, mechanical ventilation, handling and stress of the infant. Notably, the use of inotropic medications was not associated with higher BPV in our study.

In adult hypertension, high BPV is associated with end organ damage independent of absolute blood pressure values [8]. Animal studies have also shown that antihypertensive medications that buffer the enhanced BPV commonly found in hypertensive patients reduce organ injury [25]. Therefore, treatment strategies to reduce BPV may possibly be an equally important target of antihypertensive treatment in adults, as lowering of BP itself [26]. In contrast, the potential importance of BPV on end organ injury has not been investigated in the preterm infants. Our results suggest there may be value in monitoring BPV in sick preterm infants with the aim of identifying its role in cerebral injury.

An advantage of our application of frequency domain analysis is that it allows analysis of Coh and BPV in the very low frequency range. Since the amplitude of spontaneous MABP variations increase as their frequency decreases, significant disturbance in cerebral perfusion are more likely at these very low frequencies. A potential limitation of the coherence and transfer function analysis is the assumption that the MABP-TOI relationship can be modeled as a linear and stationary system [27]. By using spontaneous changes in MABP, which are of relatively smaller amplitude than induced changes often used in experimental studies, we minimized the contribution from nonlinear responses. Biological signals such as TOI and MABP are expected to have some periodic fluctuations leading to a loss of signal stationarity [9], so reducing Coh and G values in some infants. Also, temporal variations in autoregulatory capacity in an infant may lead to variations in Coh values. Our study, by using the correlation between the highest or maximum Coh with BPV, minimized the effect of the variations in Coh values caused by technical limitation of cross spectral analysis.

## Conclusion

Our study has demonstrated that in very preterm infants high blood pressure variability may easily exceed cerebral autoregulatory capacity, thereby generating significant fluctuations in cerebral oxygenation. The most critically sick infants exhibit blood pressure-dependent variations in cerebral oxygenation even with relatively low blood pressure variability, placing them at greater vulnerability to cerebral injury arising from blood pressure fluctuations.

Our study suggests potential value of continuous monitoring of TOI and BPV, combined with spectral analysis, in clinical evaluation of sick preterm infants. This evaluation may identify those preterm infants who are at greatest risk of cerebral injury, guide clinical decision-making in relation to blood pressure management, and help develop strategies to minimize fluctuations in cerebral perfusion.
